# Highly restricted specificity of aberrant exons in a saturated background of differentially expressed exons

**DOI:** 10.1016/j.gendis.2026.102130

**Published:** 2026-03-05

**Authors:** Urmi Das, Hai Nguyen, Jian Yang, Samuel Ogunsola, Chenmin Mao, Madeleine Hamilton, Ling Liu, Wenguang Cao, Chunyu Liu, Jiuyong Xie

**Affiliations:** a Department of Physiology & Pathophysiology, University of Manitoba, Winnipeg, Manitoba R3E 0J9, Canada; b Department of Biostatistics, Boston University School of Public Health, Boston, MA 02118, USA

Differentially expressed exons (DEXs), produced from alternative splicing, alternative transcription start, or alternative polyadenylation, contribute greatly to the transcriptomic and proteomic diversity.^1^^,^^2^ Dysregulation of exon usage generates aberrant exons in diseases,^3^ but their specificity and relationship to the DEXs in a whole-body background remain unknown, particularly in cancer.^4^ Here, we sought to approximate the whole-body condition by obtaining a saturated DEX list from analyzing 56 human tissue types to show that at least 99% cancer-specific exons by paired normal tissue comparisons are DEXs of a DEX-dominated normal human exome. Those associated with somatic single nucleotide variants (SNVs) are truly cancer-specific in this background, potentially generating novel peptides and cancer-specific antigens.

To estimate the total number of DEXs in the human exome, we examined the RNA-sequencing data of 56 types of nominally normal tissues (53 adult plus 3 fetal types; Fig. S1A and S1B; supplementary materials and methods) in the NCBI SRA and dbGaP databases. In the DEXSeq analysis of DEXs with all possible pairs of 56 tissue types (*n* = 1540 pairs), Ensembl-annotated exons were divided by their boundaries among different transcripts into unique bins (also called exons hereafter). In total, 370,559 exons from 27,463 genes with a minimum average of 20 normalized counts per base (exon base mean) were included, as the reference exome for subsequent analyses.

Interestingly, 357,681 exons exhibited significant differences (adjusted *p* < 0.05) in at least one of the tissue type pairs. More stringent filtering with our reverse transcription-PCR-validated protocol resulted in 327,752 exons as DEXs (88%; Fig. 1A; Fig. S1B). Increasing the exon base mean threshold up to 1000 obtained similar percentages (88.57% ± 0.83%, mean ± standard error of the mean, *n* = 14). Importantly, the predicted ratios of exon usage between tissue types correlated with reverse transcription-PCR results at a Pearson coefficient of 0.7, with 90% DEX validation (Fig. 1A; Fig. S1C). In addition, an estimated 90% of the DEXs are included in at least 10% (in molar ratio) of their gene transcripts. Moreover, the 349,833 annotated non-overlapping human exons in Ensembl release 93 had only 15,990 (4.5%) with the same chromosome coordinates in all transcripts, likely as constitutive exons. Therefore, it is likely that the majority of human exons are DEXs rather than constitutive exons, even with the validation rate and inclusion levels considered (72%).

**Figure 1:**
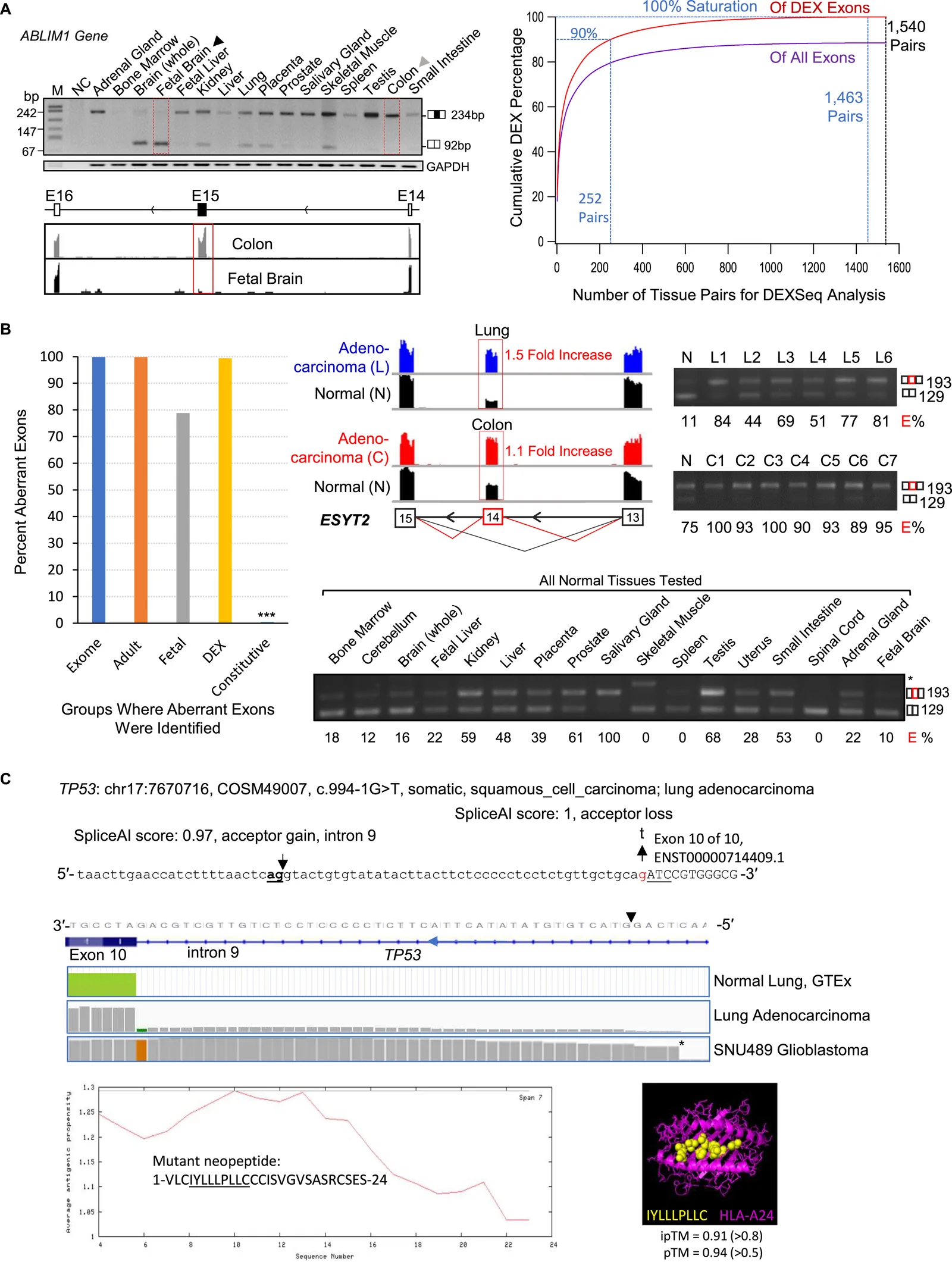
Abundance of differentially expressed exons (DEXs) (A) or aberrant exons of adenocarcinomas (B) in the human exome, and cancer-specifically expressed intron regions associated with somatic single nucleotide variants (SNVs) (C). **(A)** An example of semi-quantitative reverse transcription-PCR validation of predicted DEXs (left panel), and their saturation curve (right panel) relative to the total number of DEXs (*n* = 327,752) or all exons (*n* = 370,559) of 56 types of human normal tissues. **(B)** Left, Percent distribution of cancer aberrant exons relative to their total among different exon groups (left panel) and an example of reverse transcription-PCR validation of the ectopic expression of aberrant exons in normal tissues (right panel). E: aberrant exon 14 of *ESYT2*. **(C)** An example of cancer-specific, aberrantly expressed intron regions associated with SNVs: a somatic *TP53* variant associated with inclusion of an intronic fragment (top), RNA-sequencing evidence from tumors carrying the variant T (middle), and antigenicity of the in-frame translated novel peptide and its AlphaFold3-predicted epitope interaction with the HLA supertype A24 (bottom). ∗: extra 4 nt same as the upstream exon end, likely misassigned here in the IGV.

The accumulating number of unique DEXs increased exponentially with increasing pairs of tissue types (Fig. 1A). The number reached 90% of the total at 252 pairs, 99% at 868 pairs, and 100% at 1463 pairs, without further increase from the additional 77 pairs (Note: 54 tissue types in GTEx yield 1431 pairs only). Thus, the pairwise search has saturated the DEXs in the 56-tissue type exome.

Summaries of the DEX distribution by exon types, expression mechanisms, and tissue types are in Figure S2 and S3.

We then analyzed the adenocarcinoma RNA-sequencing data from five organs (lung, colon, pancreas, prostate, and breast) (Fig. S4), well known for their worldwide high cancer incidence and mortality, of 184 patients from the Cancer Genome Atlas (TCGA). Comparison with their paired normal tissues using DEXSeq identified 167,015 unique, differentially expressed (aberrant) exons of 13,638 genes with at least 1.1-fold change (adjusted *p* < 0.005). Surprisingly, 99.8% of them were among the reference exome (Fig. 1B; Fig. S5–S7), of which 99.9% in the adult and 78.9% in the fetal tissues. Moreover, 99.4% of them were among the DEXs (a 27-fold enrichment), and only about 0.5 % among the constitutive exons.

A similar distribution of the aberrant exons, 95.8% in the DEX and 99.9% in the reference exome, was also found in an independent dataset of 10k “cancer-specific” exons against a background of 31 normal tissue types,^5^ corresponding to the 456 tissue type pairs at 95.4% DEX saturation (Fig. 1A).

Similar examination by focusing on 14,523 aberrantly increased intron regions (AIIR, by edgeR, ≥ 4 folds, FDR ≤ 0.01) identified 69% of them also in the 56 normal tissue types (Fig. S8 and S9). However, the remaining 31% AIIRs could provide more cancer-specific sequences, particularly those associated with SNVs. We thus first filtered the AIIRs for their association with TCGA or COSMIC SNVs (FATHMM pathogenic score > 0.7) and examined them in the data of 184 adenocarcinoma patients (Fig. S10). We identified 57 intron variants from 110 SNVs among 109 patients. Cancer-specific aberrantly expressed intron regions were observed in the IGV of the corresponding RNA-sequencing data for thirteen variants in six genes: *TP53*, *VHL*, *PTEN*, *ACVR1B*, *STK11*, and *SMARCA4* (Table S1; Fig. S11 and S12), but not in any of the 56 types of normal tissues, supporting their specificity in the reference exome background.

We then used SpliceAI (> 0.8) with 7085 unique pathogenic SNVs (FATHMM > 0.5) to identify more aberrant exons in association with 313 unique variants (91% somatic; Table S2; Fig. S13 and S14), including eleven of the above thirteen. Sixteen of them were substitution or deletion/frameshift variants that were not marked as splice variants in the databases. The majority of them (260) have no effects described. Of the host genes, tumor suppressors *TP53* (95), *PTEN* (18), *RB1* (15), *STK11* (13), *NF1* (9), and *ATM* (8) harbor about 50% of the 313 variants, implying them as a major group of targets among the splicing-inducing, pathogenic SNVs in tumorigenesis.

Examples of the predicted effects on intron sequence usage upon cancer-specific aberrant splicing or alternative transcription start/alternative polyadenylation by representative SNVs are in Figure S13B and Table S2. None of these expressed intronic sequences was found in the 56 tissue-type RNA-sequencing data. Moreover, the potential of such authentic cancer-specific sequences with predicted HLA-binding epitopes for cancer vaccine or immunotherapy is illustrated with the *TP53* c.994-1G>T variant (Fig. 1C), one of 29 epitopes (27 of TP53) identified from the RNA-sequencing data of MutSpliceDB.

In conclusion, our analysis supports that DEXs, instead of constitutive exons, dominate the human exome. In such a background, at least 99% aberrant exons in cancer are ectopically expressed normal DEXs, emphasizing the necessity of broader transcriptomic coverage for cancer-specific exons in whole-body applications. Moreover, integration of genetic variant data identifies authentic cancer-specific intronic regions as novel targets potentially for diagnosis, immunotherapy, or vaccination. Together, these insights also advocate for a more thorough understanding of exon usage and splicing regulation in both health and disease to pave the way for more precise and safe holistic therapeutic strategies.

## CRediT authorship contribution statement

**Urmi Das:** Writing – review & editing, Writing – original draft, Visualization, Validation, Project administration, Methodology, Investigation, Formal analysis, Data curation, Conceptualization. **Hai Nguyen:** Software, Formal analysis, Data curation. **Jian Yang:** Investigation, Formal analysis. **Samuel Ogunsola:** Investigation. **Chenmin Mao:** Investigation. **Madeleine Hamilton:** Methodology, Investigation. **Ling Liu:** Validation. **Wenguang Cao:** Validation. **Chunyu Liu:** Supervision. **Jiuyong Xie:** Writing – review & editing, Visualization, Supervision, Resources, Investigation, Funding acquisition, Formal analysis, Conceptualization.

## Ethics declaration

Research using the human tissue or genome/transcriptome data was approved by the Research Ethics Board of the University of Manitoba (#HS21726). Informed consent from the patients was obtained by the TCGA-participating groups or the Ontario Tumor Bank.

## Funding

This work was supported in part by the 10.13039/501100000038Natural Sciences and Engineering Research Council of Canada (No. RGPIN-2016-06004, RGPIN-2022-05023) and a Manitoba Research Chair fund to Jiuyong Xie.

## Conflict of interests

The authors declared no conflict of interests.
