# Supplementary material for: Highly restricted specificity of aberrant exons in a saturated background of differentially expressed exons
Source: Genes Dis. 2026 Mar 5;14(2):102130. doi: 10.1016/j.gendis.2026.102130 (PMC13651660; doi:10.1016/j.gendis.2026.102130)
Supplement: Supplementary file 1 [file mmc4.pdf]

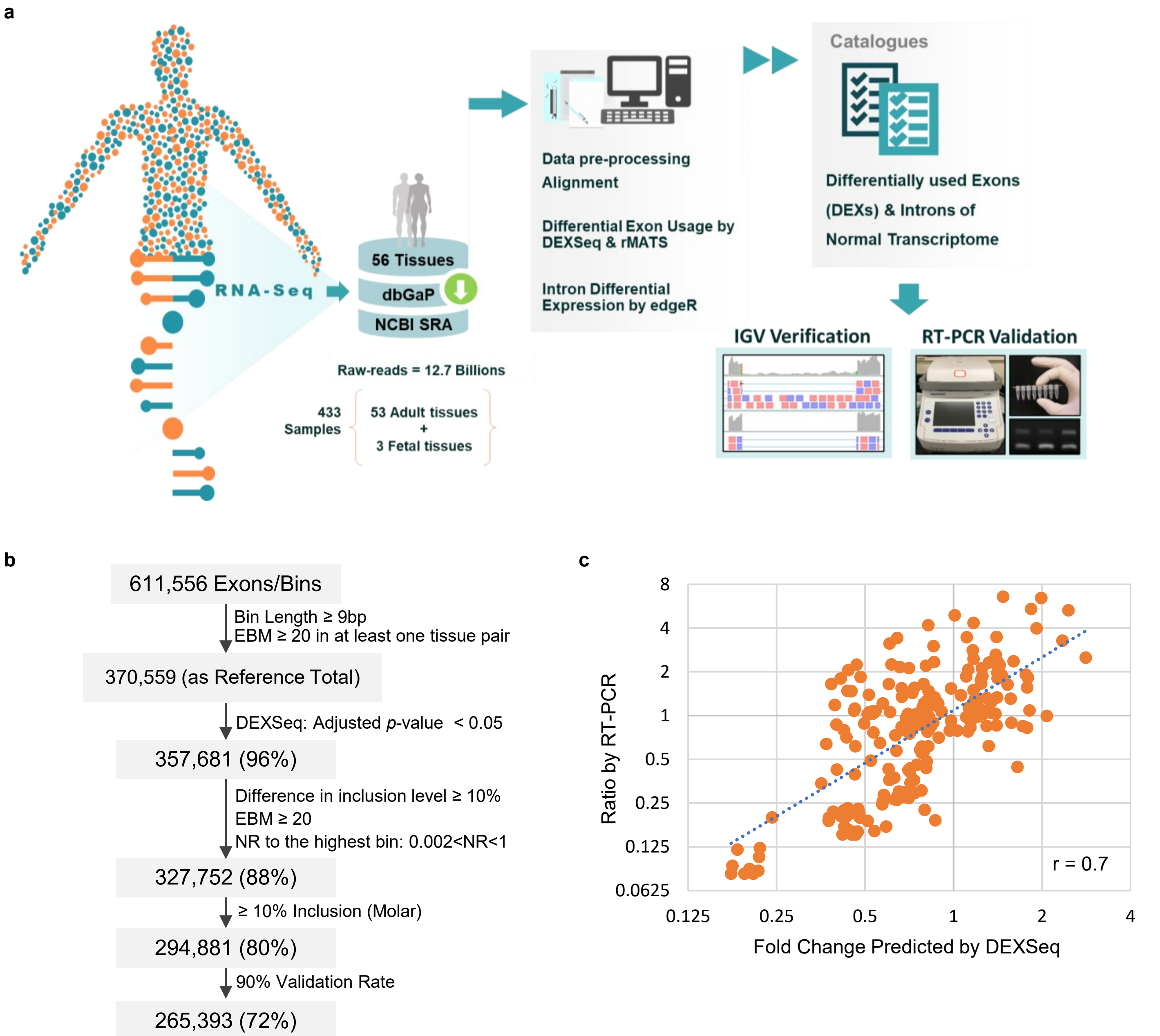

Supplementary Figure 1. a. Study overview. **b.** Flowchart of our filtering pipeline for DEX usage analyses in 56 types of nominally normal human tissues. NR: ratio of normalized reads. **c.** Correlation of predicted fold change (X-axis) with the ratio of exon inclusion percentages of RT-PCR products (Y-axis) between samples (in log2 scales, n = 240 alternative splicing events of 12 genes in 110 tissue pairs).

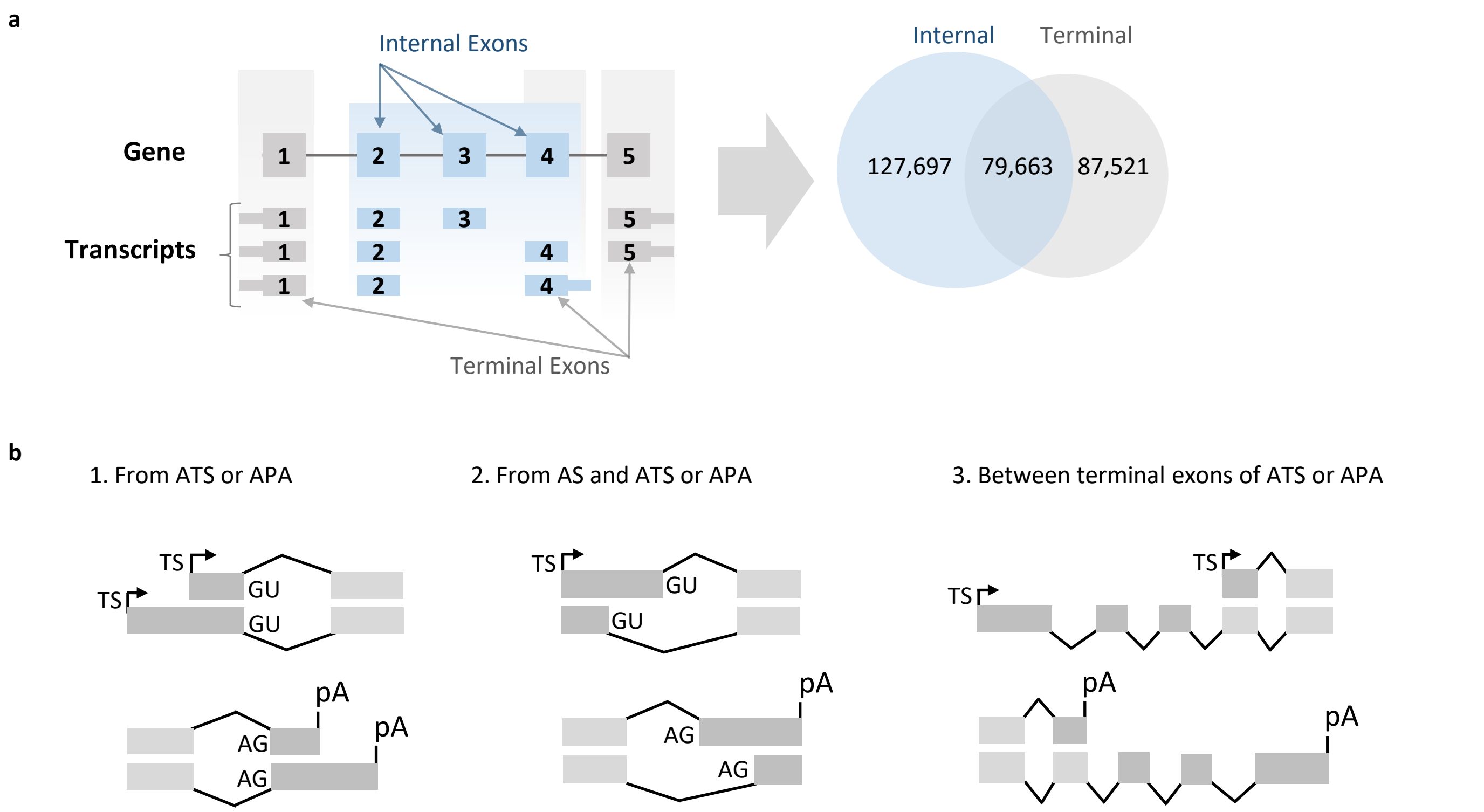

Supplementary Figure 2: Diagrams of DEXs produced from AS, ATS or APA.

a. DEXs as internal or terminal exons. **b.** Representative patterns of DEXs produced from ATS or APA with or without AS (57% in total), with the rest 43% from AS only, by random examination of three subsets of DEXs (n = 100 each). The 3rd group comprises 53% of all ATS/APA-induced DEXs.

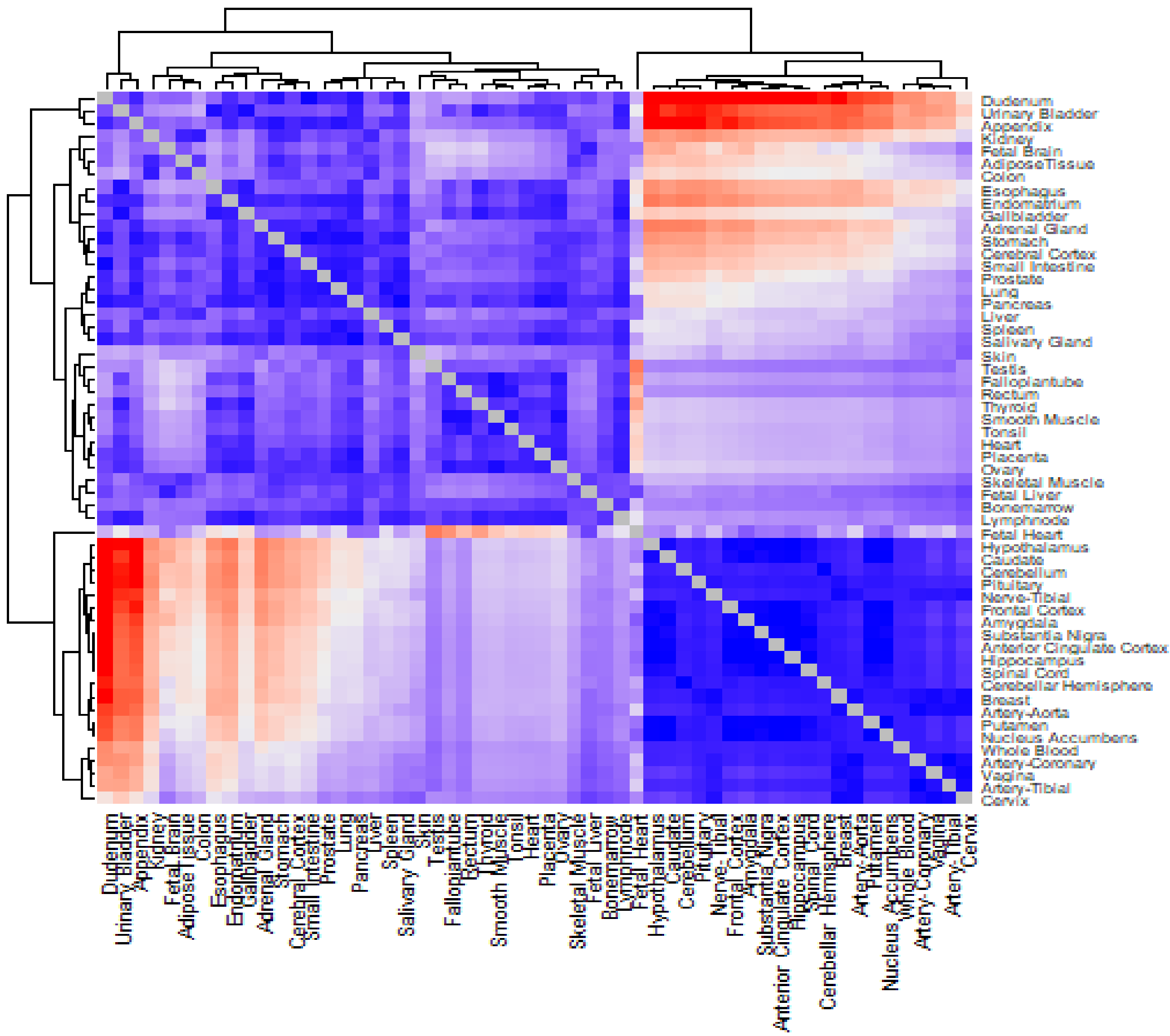

Supplementary Figure 3: Global distribution of DEXs across tissues. Symmetrical heatmap of pair-wise correlation among 56 tissues based on the number of DEXs. The brain regions, when compared with other tissues including duodenum, urinary bladder, appendix, kidney, esophagus, and endometrium, showed much higher variabilities yielding more DEXs, than among the brain regions themselves. Higher variabilities were also observed between the fetal and adult tissues.

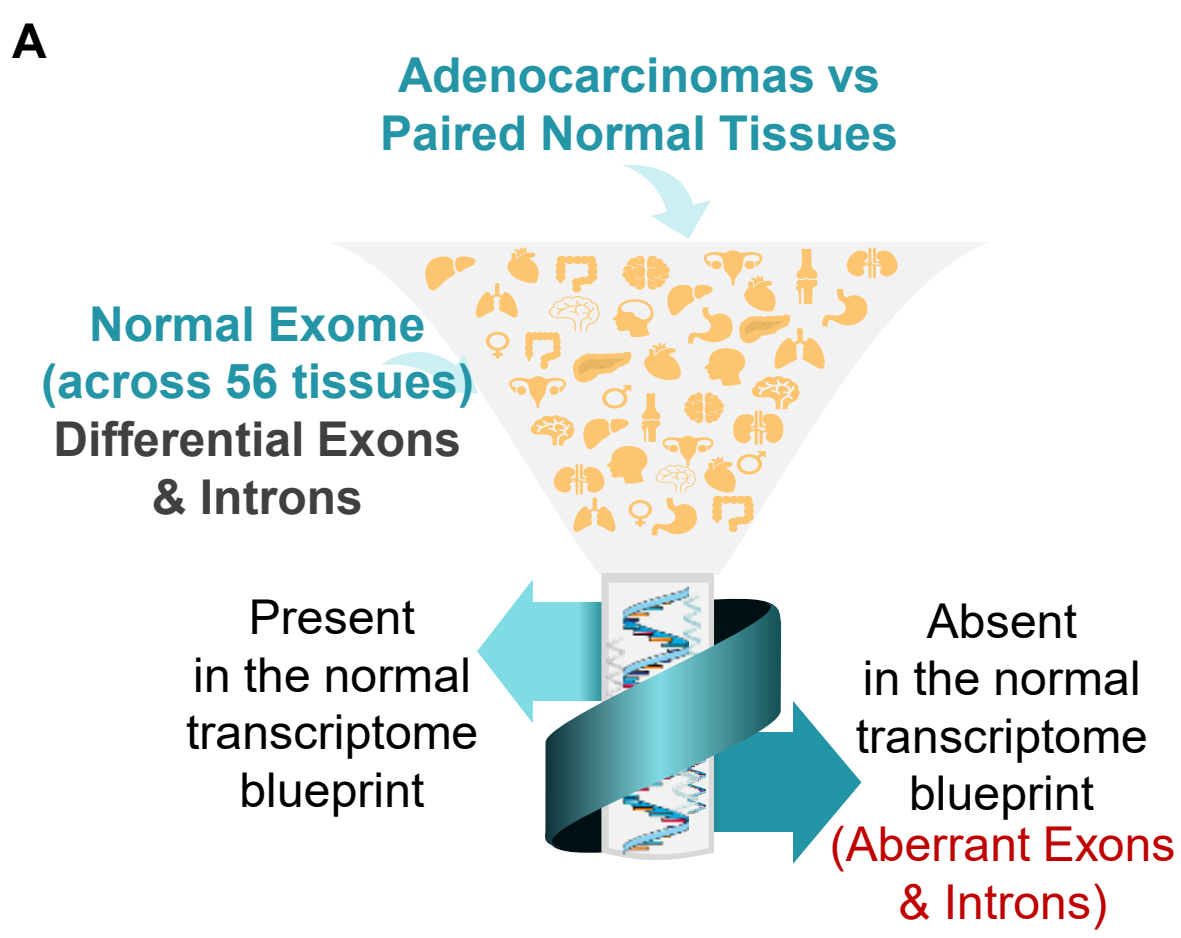

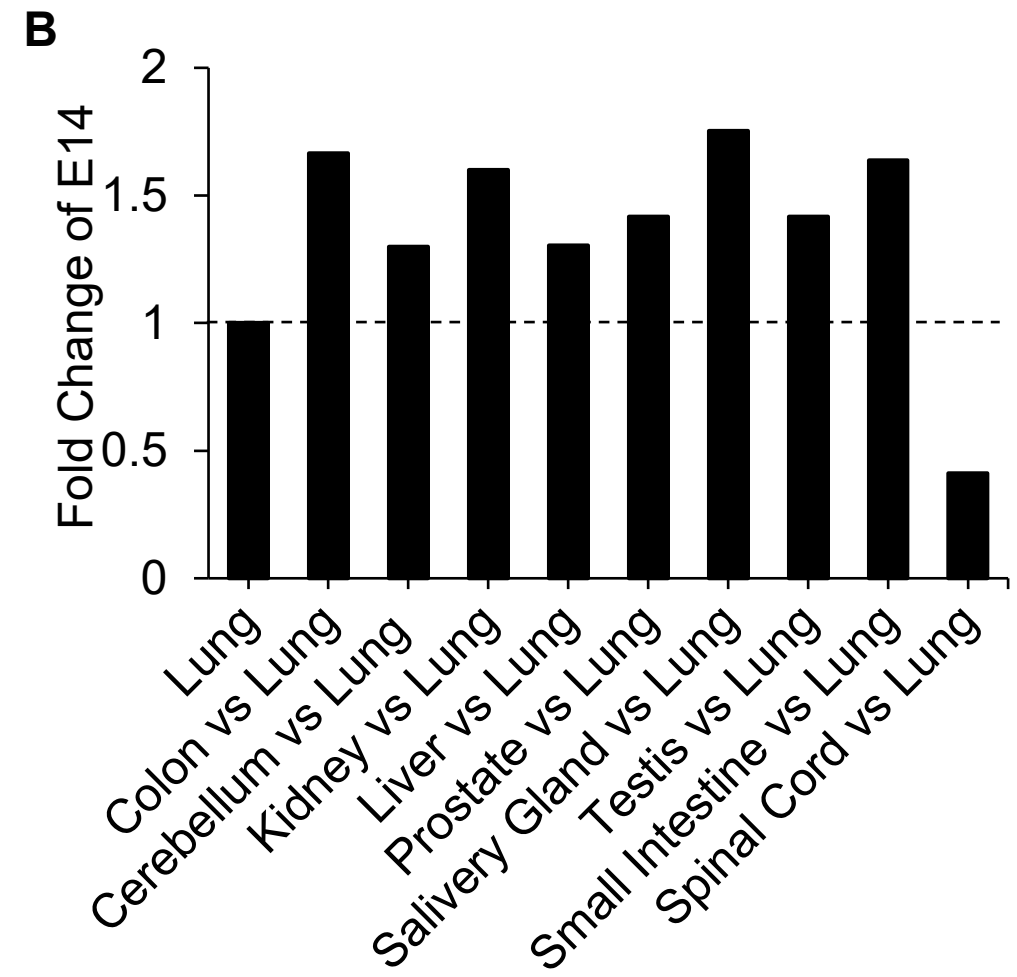

Supplementary Figure 4. **Ectopic expression of aberrant exons in adenocarcinomas. A**. An overview of cross-checking the aberrations in adenocarcinomas against the normal reference exome. **B**. An example of aberrantly increased exons: E14 of *ESYT2* gene in lung adenocarcinoma (Fig. 1B). It is also predicted to be increased in a number of other normal tissues as indicated, compared to normal lung by DEXSeq analysis.

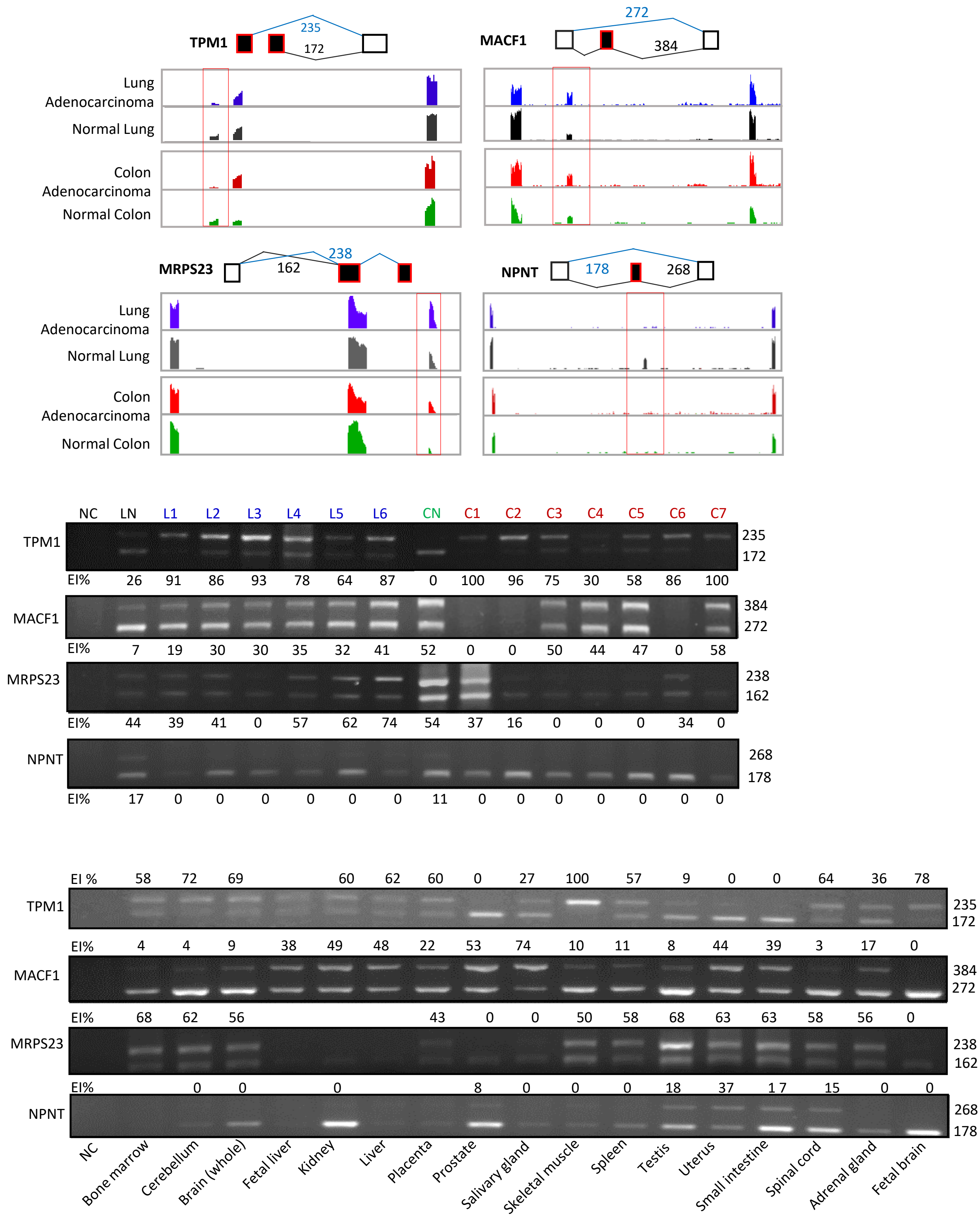

Supplementary Figure 5: Validation of ectopic exon usage of four genes- *TPM1* (Exon 2), *MACF1* (Exon 99), *MRPS23* (exon 1), *NPTN* (Exon 4) in lung and colon adenocarcinoma patients. EI%- Exon inclusion percentage, NC-Negative control, LN- Normal Lung, (L1-L6) 6 Lung adenocarcinoma patients, CN-Normal colon, (C1-C7) 7 Colon adenocarcinoma patients. Increased usage of these exons is also shown in the RT-PCR result of 15 different adult and 2 fetal tissues (bottom panel).

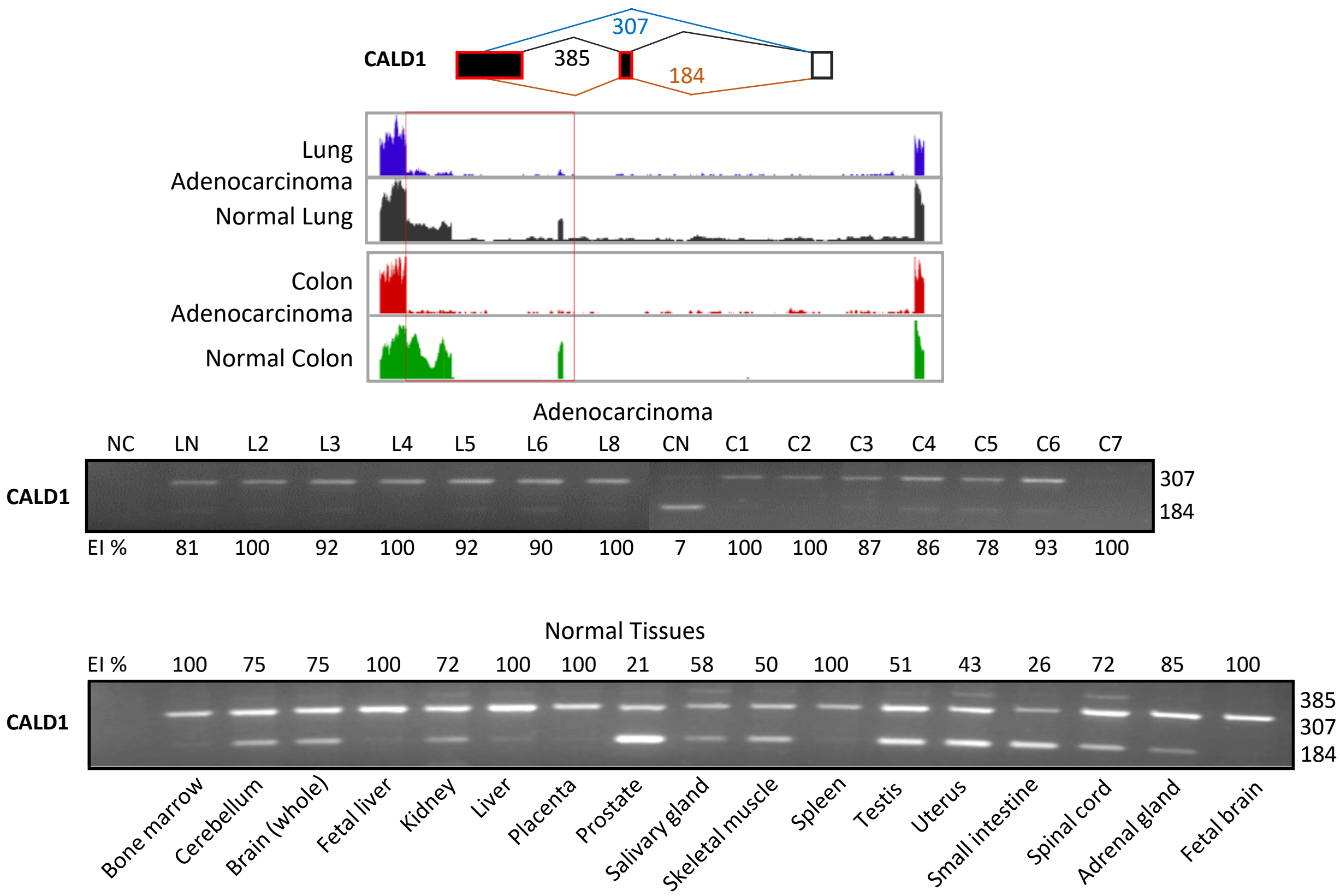

Supplementary Figure 5b: Validation of ectopic exon usage of CALD1 (Exon 4) in lung and colon adenocarcinoma patients. The differential splicing of it is also shown in 15 different adult and 2 fetal tissues. EI%- Exon inclusion percentage, NC-Negative control, LN- Normal Lung, (L1-L6) 6 Lung adenocarcinoma patients, CN-Normal colon, (C1-C7) 7 Colon adenocarcinoma patients.

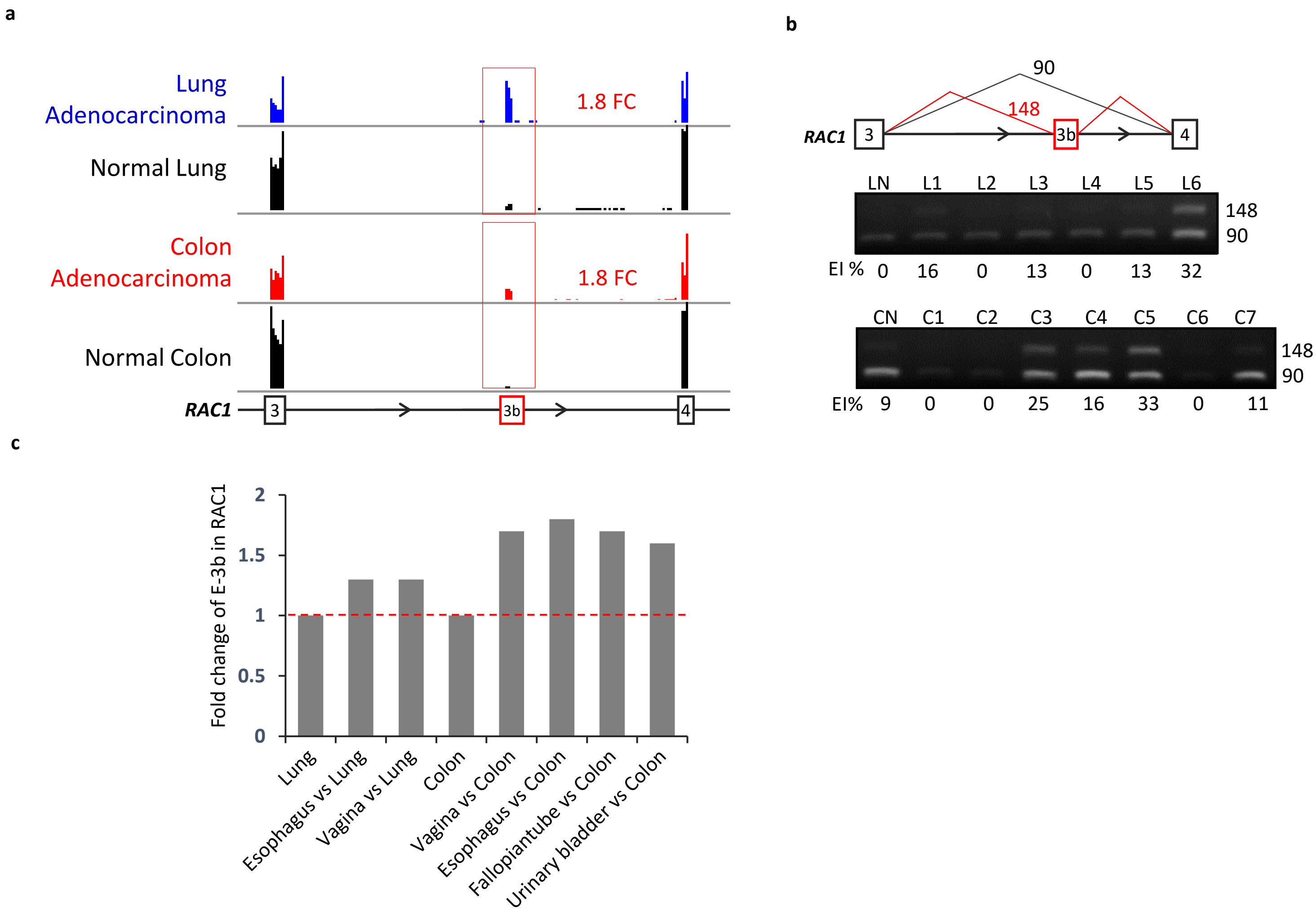

Supplementary Figure 6: Ectopic exon usge of exon 3b of RAC1 in adenocarcinoma. a. IGV view of 'cancer-specific' exon E-3b of RAC1 with fold change by DEXSeq **b.** E-3b inclusion in adenocarcinoma validated by RT-PCR **c.** E-3b inclusion in other normal tissues compared to normal lung and colon with fold change by DEXSeq.

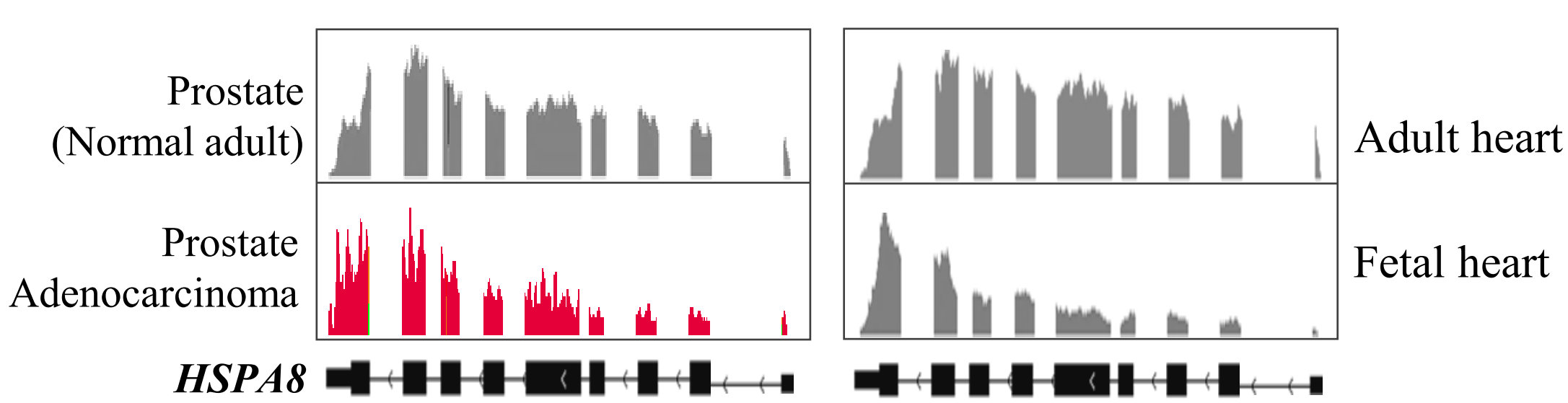

Supplementary Figure 7: Developmental stage-specific similarities of *HSPA8* dysregulation between tissues. The *HSPA8* gene transcript processing in prostate adenocarcinoma exhibits similar patterns as in normal fetal but not adult heart tissue samples.

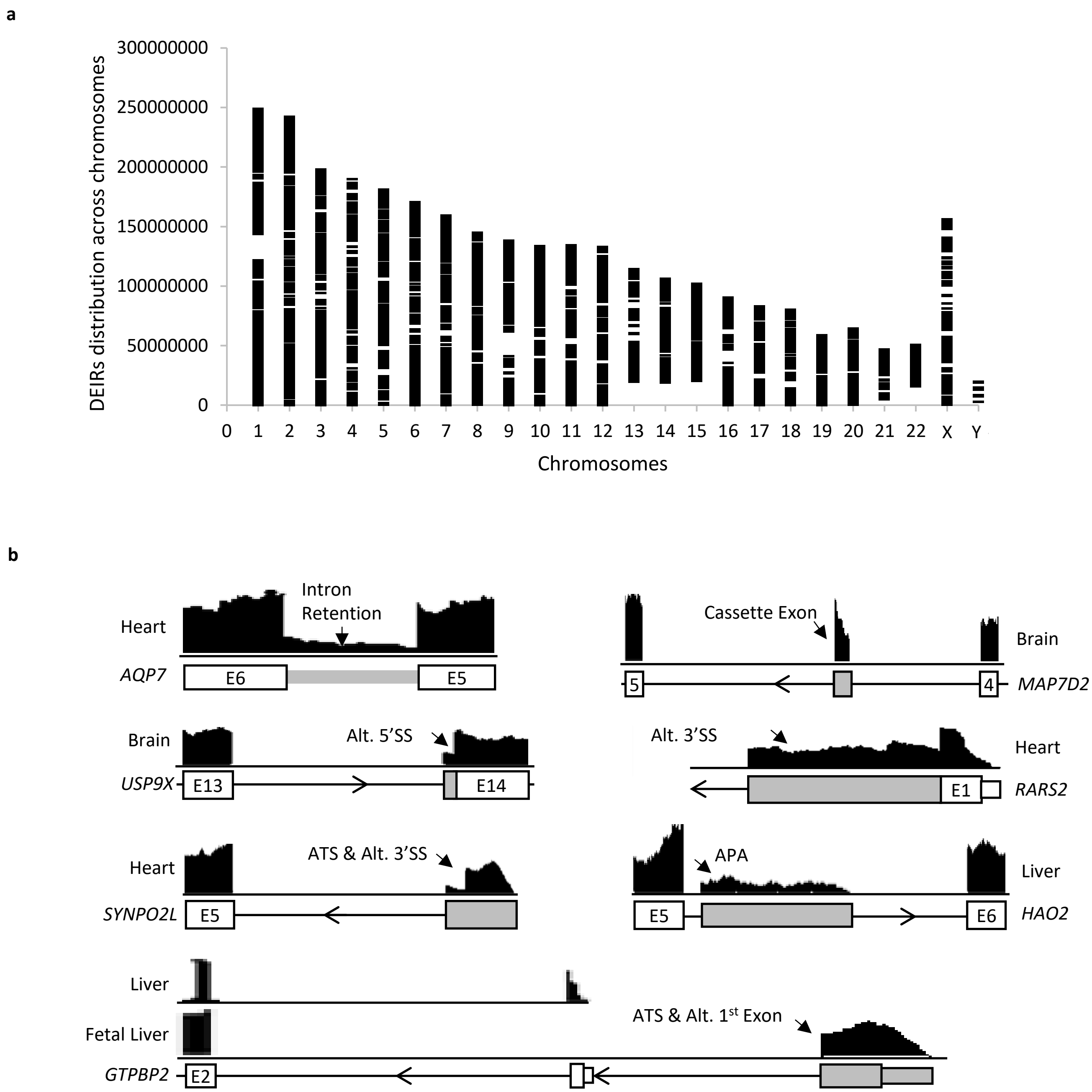

Supplementary Figure 8: Differentially expressed intron regions (DEIRs) of the normal transcriptome across 56 different tissues. a. Distribution of the total DEIRs by their chromosome locations. **b.** Individual examples of events identified as DEIRs in gene AQP7 (Aquaporin-7), MAP7D2 (MAP7 Domain Containing 2), USP9X (Ubiquitin Specific Peptidase 9 X-Linked), RARS2 (Arginyl-TRNA Synthetase 2, Mitochondrial), SYNPO2L (Synaptopodin 2 Like), HAO2 (Hydroxyacid Oxidase 2) and GTPBP2 (GTP Binding Protein 2).

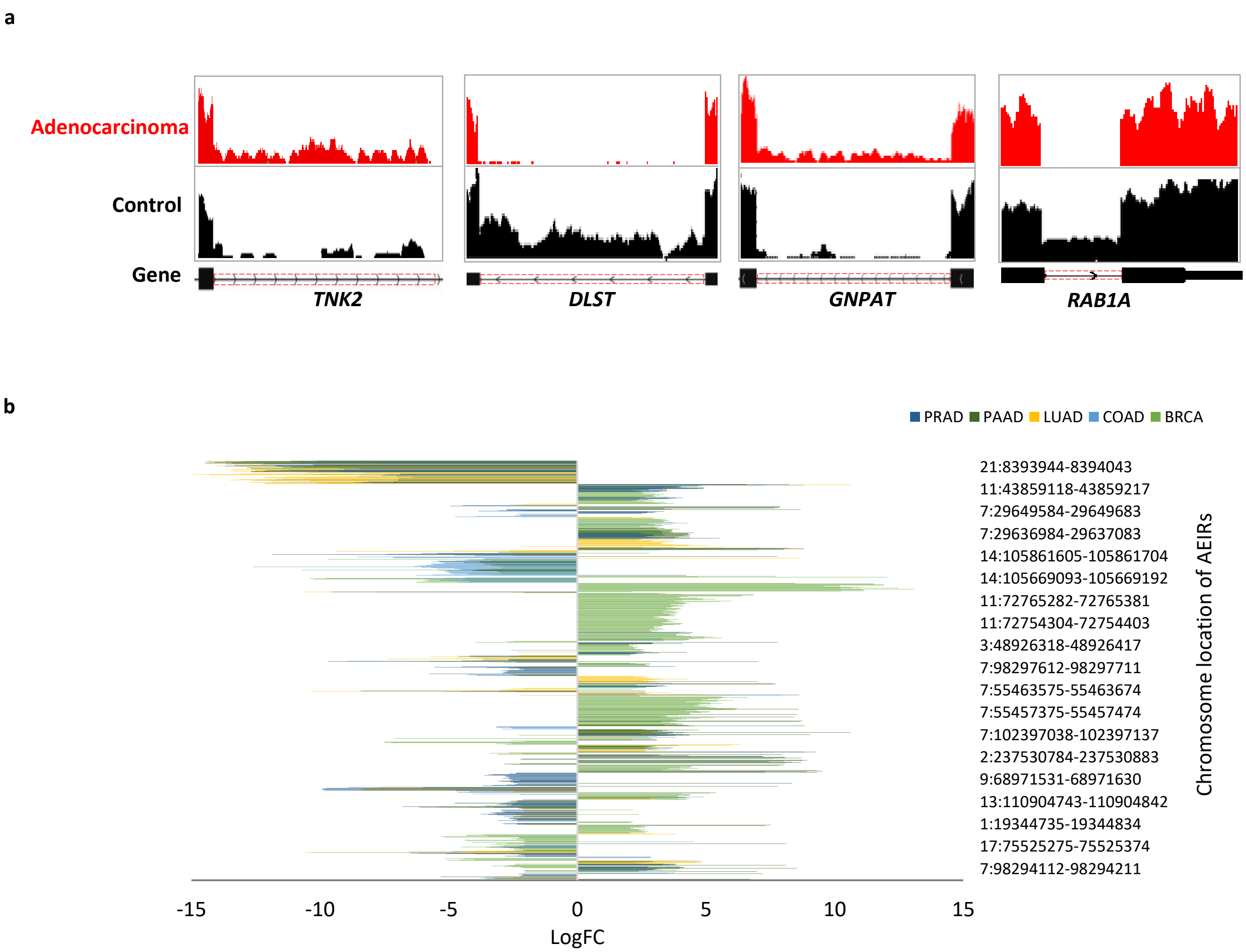

Supplementary Figure 9: Aberrantly expressed intron regions (AEIRs) in adenocarcinoma. a. Individual examples of AEIRs identified in gene *TNK2* (Tyrosine Kinase Non-Receptor 2), *DLST* (Dihydrolipoamide S-Succinyltransferase), *GNPAT* (Glyceronephosphate O-Acyltransferase), *RAB1A* (Member RAS Oncogene Family) shown in IGV. **b.** AEIRs of 33 common genes in lung, colon, prostate, pancreatic and invasive breast adenocarcinoma. The X-axis represents Log2fold change of the intron regions compared to their paired normal and the Y-axis represents the intron locations. Aberrantly increased intron regions (**AIIRs**) can be seen clearly in some of the examples here in both **a** and **b**.

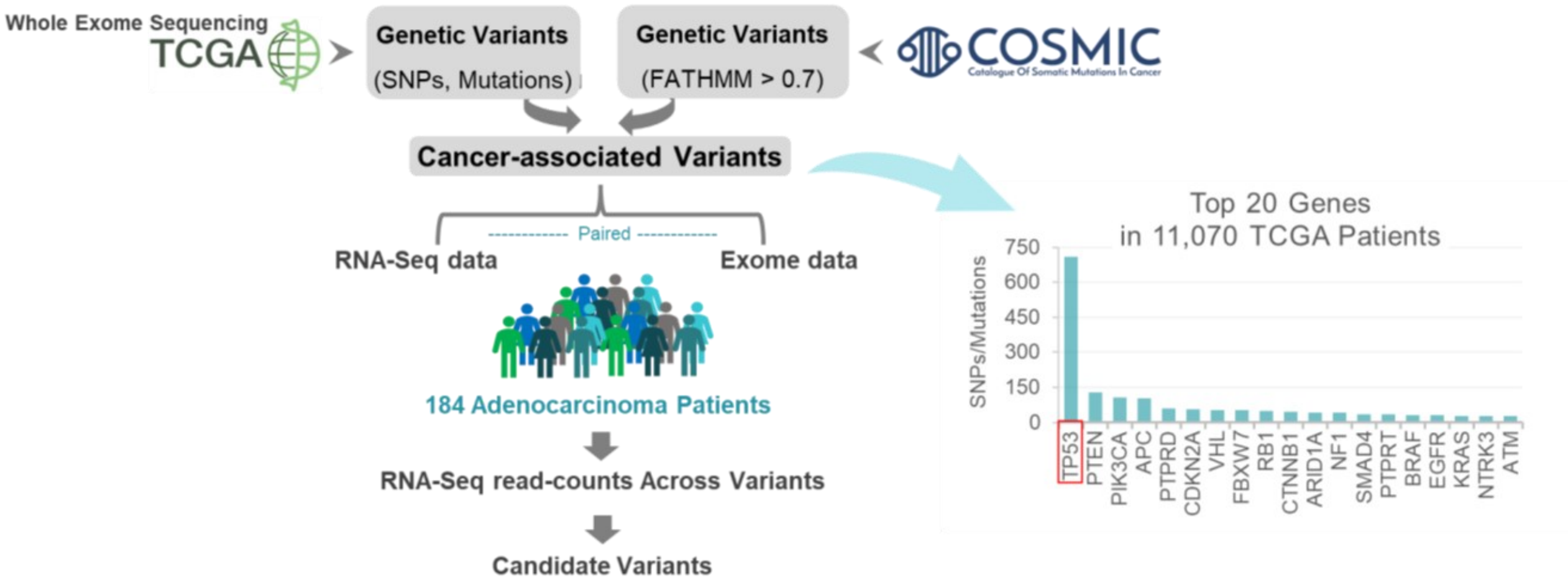

Supplementary Figure 10. Filtering for pathogenic SNVs from cancer databases. An overview of filtering process pathogenic genetic variants from WGS to verify SNV-associated splicing aberrations by the paired RNA-seq data, with the top 20 ranked mutated genes in the bar graph.

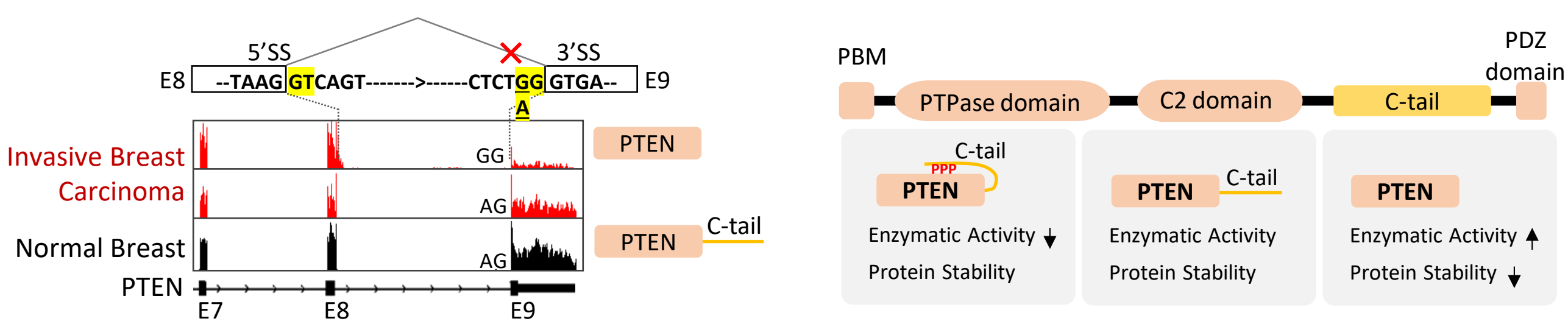

Supplementary Figure 11: An SNV-associated 'cancer-specific' splice variants. The IGV view of *PTEN* gene in patients of breast carcinoma with (top panel) or without (middle panel) A>G substitution in the 3'SS of terminal exon 9. The A>G substitution leads to transcript shortening at the upstream 5′SS of exon 8. The resulting protein lacks the amino acids 353–403 containing a C-tail. At the bottom, is a diagram of domains of PTEN protein. The C-tail phosphorylation status regulates for PTEN activity and stability where the protein without the C-tail is more active but less stable.

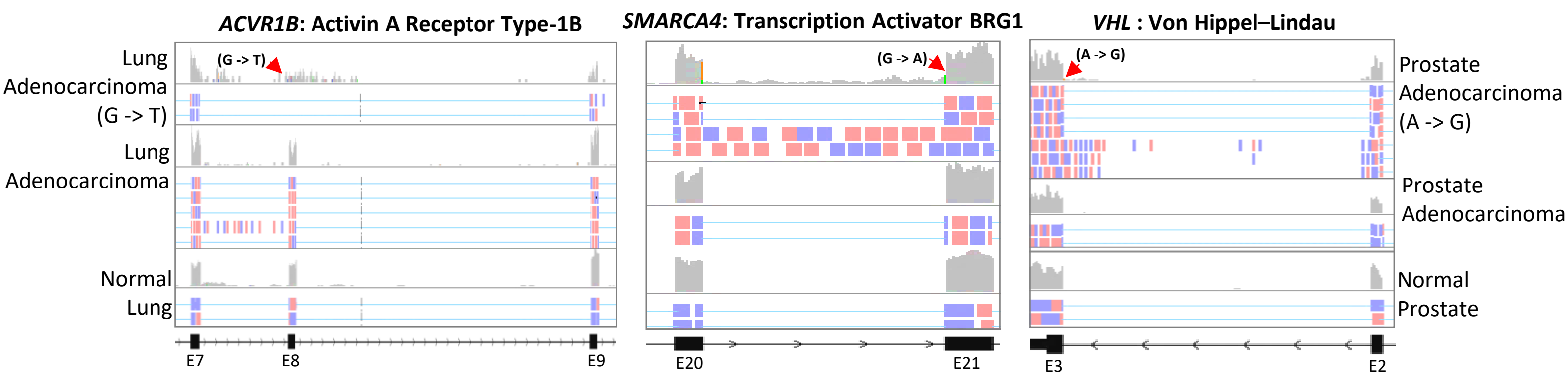

Supplementary Figure 12: SNV-associated AIIRs *in genes ACVR1B, SMARCA4* and *VHL,* verified by RNA-Seq data of adenocarcinoma patients.

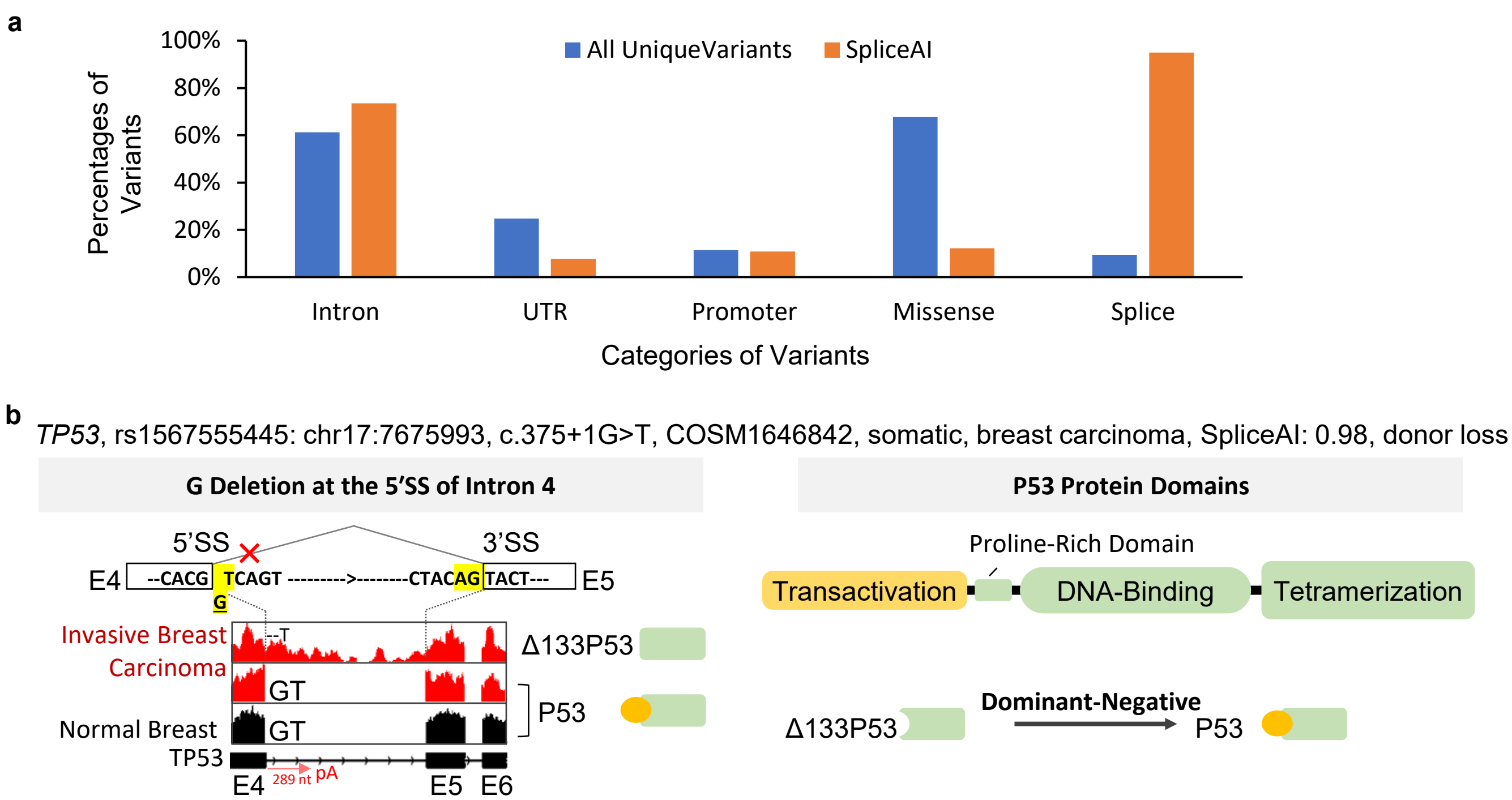

Supplementary Figure 13. Identified pathogenic SNVs associated with authentic cancer-specific exons or aberrantly increased intron regions (AIIR) from SpliceAI analysis. a. Distribution of unique variants in the starting pool and the SpliceAI > 0.8 group. **b**. Novel exon fragments generated after the abolishment of canonical splice site by a SNV. The IGV view of *TP53* in breast carcinoma patient with (top panel) or without (middle panel) delG in the 5′SS. Deletion of G in 5′SS of exon 4 likely lead to the use of ATS at exon 5 (NM_001407266.1) or intron retention. The resulting transcripts, when translated, generate N-terminally truncated p53 proteins lacking the transactivation domain. On the bottom, is a diagram of the domains of protein p53. P53 without transactivation domain acts as dominant negative inhibitor of its wild type. *: extra read peaks of TCAG, same as the exon 9 end, likely misaligned.

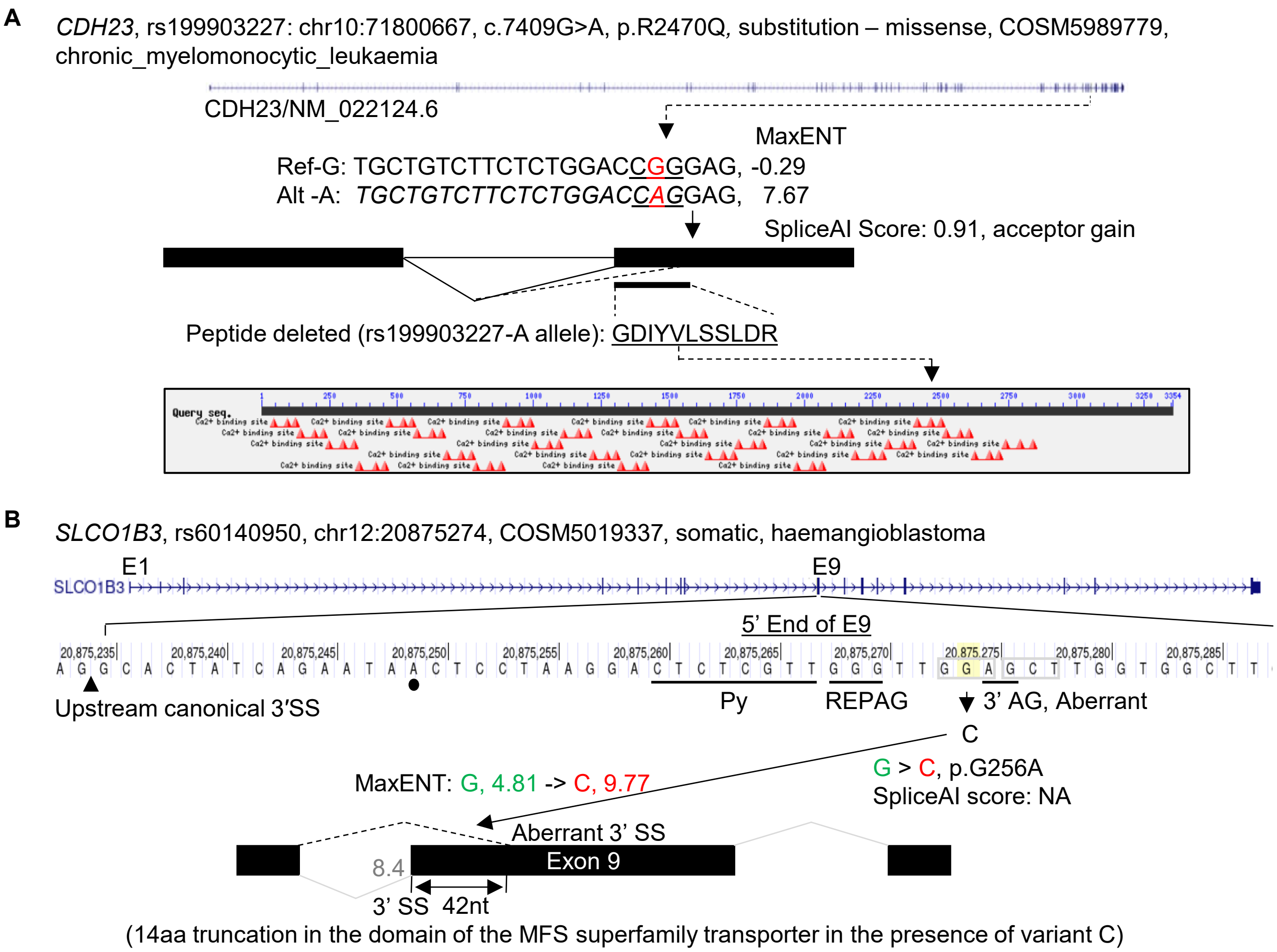

Supplementary Figure 14: Additional examples of cancer-specific variants resulted from SpliceAI-predicted SNVs. A. A SNV originally identified as missense was predicted by SpliceAI to cause skipping of an exon fragment and peptide deletion in important protein domains in cancer. Shown is the rs199903227 in a 3′ end exon of the *CDH23* gene. The deleted peptide is within a Ca^{2+}-binding site at the COOH-terminal of the cell adhesion protein, a suppressor of cancer metastasis. **B**. A strong splice site score increase by an exonic SNV causing partial exon skipping but underestimated by SpliceAI. Shown is a GAG trinucleotide preceded by the splicing repressor GGG to be turned into a strong splice site CAG by the SNV.
