# Supplementary material for: Highly restricted specificity of aberrant exons in a saturated background of differentially expressed exons
Source: Genes Dis. 2026 Mar 5;14(2):102130. doi: 10.1016/j.gendis.2026.102130 (PMC13651660; doi:10.1016/j.gendis.2026.102130)
Supplement: Supplementary file 2 [file mmc1.docx]

**Materials and Methods**

### Data Pre-processing

RNA-Seq data of 56 types (53 adult and 3 fetal) of nominally normal human tissues were obtained from the NCBI SRA and dbGaP databases, originally contributed by three sources: 32 adult tissue types from a project (PRJEB6971) of the Science for Life Laboratory, Royal Institute of Technology, Stockholm (adipose tissue, adrenal gland, appendix, bone marrow, cerebral cortex, colon, duodenum, endometrium, esophagus, fallopian tube, gallbladder, heart, kidney, liver, lung, lymph node, ovary, pancreas, placenta, prostate, rectum, salivary gland, skeletal muscle, skin, small intestine, smooth muscle, spleen, stomach, testis, thyroid, tonsil, urinary bladder) [1]; 21 from the Genotype-Tissue Expression (GTEx) (Accession number: phs000424.v7.p2) project of the National Institutes of Health (NIH) (amygdala, anterior cingulate cortex, aorta, breast, caudate, cerebellar hemisphere, cerebellum, cervix, coronary artery, frontal cortex, hippocampus, hypothalamus, nucleus accumbens, pituitary gland, putamen, spinal cord, substantia nigra, tibial artery, tibial nerve, vagina, whole blood) [2] and three types of fetal tissues from a project (PRJNA268504) of the Peking University (whole brain, heart and liver) [3].

In total, we obtained 12.7 billion of paired-end raw reads (70-100bp) from 433 tissue samples of 56 normal tissue types. The quality control measurements were performed by FastQC and Trimmomatic[4] and we aligned the processed, high-quality reads (35-90bp) to the human transcriptome (Ensembl GRCh38.78) allowing maximum 2 mismatches by TopHat 2.1.1[5] resulting in 8.8 billion of uniquely mapped reads as high quality concordant pairs for subsequent analyses (about ~157 millions of reads per tissue on average).

### Annotation Preparation

For the differential exon usage analysis, the reference transcriptome (Ensembl GRCh38.78) was divided into 611,556 bins (hereafter exons) based on the exon boundaries in different transcripts. As the RNA-Seq data used in this study comprised of unstranded reads, hence the exonic regions overlapped between genes were excluded from the reference to minimize the false positive outcomes. Next, we also excluded the exons shorter than 9bp and included the exons with exon base mean (average exon read counts across samples) minimum 20 in any one of the 56 tissue type pairs and obtained the reference transcriptome comprising of 370,559 unique exons.

As we know the exon boundaries, using the exon coordinates of Ensembl GRCh38.78, we also divided the introns into bins and obtained a reference transcriptome of the unannotated intragenic regions for the intron differential expression analysis. Introns longer than 200 bp were divided into 100 bp bins.

### Pairwise Analysis of Differential Exon Usage

We have used DEXSeq [6] for differential exon usage analysis across tissue types where the ratio of read counts of the target exon to the read counts of all other exons of the same gene infers differentially used exons (DEXs) including both internal and terminal exons. Therefore, DEXSeq identifies DEXs mediated by alternative splicing (AS), alternative transcription start (ATS) or alternative polyadenylation (APA). In order to identify the maximum number of DEXs across tissue types, we have compared the tissue types pairwise in all possible combinations (C(56,2)) comprising 1,540 tissue type-pairs in total.

We have used stringent filtering criteria as reported in our previous study [7] to obtain a highly confident list of DEXs where exons with exon base mean (EBM) less than 20 (the mean of read counts across all tissue type pairs for that exon), average base mean (ABM) less than 20 (the mean of read counts across all tissue type pairs for that gene), the ratio of target exon-EBM (normalized to the exon length) to the highest EBM in that gene, less than 0.002, adjusted *p* value higher than 0.05 and fold change less than 0.1 were excluded.

In addition to DEXSeq, for a group of tissue types (35) we have also used replicate MATS (Multivariate Analysis of Transcript Splicing) [8], which yielded additional splicing events (FDR < 0.05) not overlapped with the DEXSeq outputs. With the rMATS algorithm, which measures reads spanning exon-exon junctions, we identified 50,123 additional alternative splicing events (~14% more) in 595 tissue type pairs that were often not detected by the DEXSeq method, such as the STREX exon [9, 10].

We should note that our analysis was not able to include more types of fetal tissue samples beyond the three in this study, nor examine the sex differences of DEXs[11]; therefore, the current DEX proportion of 88% should still be an underestimate.

### Tagging Terminal Exons

To estimate the relative contribution by each of AS, ATS and APA mediating DEXs, we have tagged all DEXs as whether or not terminal exons using the coordinates of different transcripts for each host gene. Since the DEXs co-mediated by AS and ATS/APA could be very complex to comprehend, we grouped the terminal DEXs considering the comparatively simpler situations as follows -

DEXs containing different transcription start sites with the same splice donor sites

DEXs containing different polyadenylation sites with the same splice acceptor sites

DEXs containing alternative splice sites with the same transcription start or polyadenylation sites

### Pairwise Analysis of Intron Differential Expression

Using our transcriptome reference of the unannotated regions, the reads mapped to intron bins were counted by FeatureCounts[12]. The intron read counts normalized to their gene level were compared between tissue types by edgeR [13] to identify the differentially expressed intron regions (DEIRs) across tissue types. We have excluded the intron regions shared by multiple genes of the same or opposite strand and further shortlisted the differentially expressed intron regions (DEIRs) with FDR ≤ 0.01 and a minimum of Log2 fold changes. Similarly as exons, the intron analyses were also performed pairwise in 1540 tissue type-pairs from 56 tissue-types. We identified 329,109 unique differentially expressed intron regions (DEIRs) of 12,975 genes (S_Fig. 7a-b) in normal tissues, and 28,007 aberrantly expressed (AEIR) from 4,448 genes in the adenocarcinomas compared to the paired normal tissues (fold change ≥ 4, FDR ≤ 0.01), with 14,523 (52%) increased in at least one of the adenocarcinomas (S_Fig. 8a).

### Validation by RT-PCR after DEXSeq Analysis

We validated 244 out of 269 alternative splicing events from 24 exons in total by semi-quantitative RT-PCR: 10 in sixteen human tissue types (adrenal gland, bone marrow, brain, colon, fetal brain, fetal liver, liver, kidney, lung, placenta, prostate, salivary gland, skeletal muscle, spleen, small intestine and testis), and 14 in GH3 cells from a separate study of the same DEXSeq filtering criteria. Human tissue RNA samples (1µg/µl) were purchased from Clontech Laboratories Inc. (Cat No. 636643)[14]. About 500ng of RNA was used for 20 µl of reverse transcription reaction. Polymerase Chain Reaction (PCR) for splicing analysis was performed with 30 cycles for different genes. PCR products were resolved in 2.5-3.5% agarose gels containing ethidium bromide and visualized with a digital camera under ultraviolet light. The percentages of exon inclusion were calculated from the band intensities and normalized by their product lengths to molar ratio.

### Functional Annotation Clustering

Functional annotation and classification of related genes were analysed using DAVID under high stringent conditions [15].

### Analysis of Adenocarcinoma Data

The RNA-Seq data of 184 patients from five types of adenocarcinomas: lung (54 patients), colon (15), pancreatic (35), prostate (36) and invasive breast carcinoma (44) were downloaded from the Cancer Genome Atlas (TCGA) database (Accession number: phs000178.v9.p8) [16] [17]. The raw data (50bp long paired-end reads) were processed similarly as the normal tissues described above. The samples of adenocarcinoma for each type were compared with their corresponding normal by DEXSeq and edgeR to identify the aberrantly expressed exons and introns in adenocarcinoma, respectively.

### Validation of Aberrant Splicing by Semi-quantitative RT-PCR

The tumor samples were purchased from the Ontario Tumor Bank. For RNA extraction, the frozen tumor samples were excised to get tissue from the tumor centre and homogenized immediately with appropriate volume of lysis buffer (with β-ME) and step-by-step tissue RNA purification protocol for Qiagen RNeasy mini kits was followed. The predicted aberrant splicing events from the above TCGA RNA-Seq analysis were validated using the extracted RNAs from the tumor samples by semi-quantitative RT-PCR similarly as described above for the normal tissues.

### Determining the expressed intron regions and antigenicity of peptide sequence

The cancer-specific nucleotide sequences of the expressed intron regions were manually verified by RNA-Seq data from cancer patients or patient-derived cell lines at the MutSpliceDB[18], translated into protein/peptide sequences in frame with the upstream coding regions. The antigenicity of the peptide regions were determined at the webserver <http://imed.med.ucm.es/Tools/antigenic.pl> [19]. The CTL epitopes of the protein/peptide sequences were determined using NetCTL 2.0 [20]. The antigenic neopeptide interaction with the HLA supertype A24 was modeled in Google AlphaFold3.0 with high confidence scores >0.9 [21].

**References**

1. Uhlen, M., Fagerberg, L., Hallstrom, B. M., Lindskog, C., Oksvold, P., Mardinoglu, A., Sivertsson, A., Kampf, C., Sjostedt, E., Asplund, A., Olsson, I., Edlund, K., Lundberg, E., Navani, S., Szigyarto, C. A., Odeberg, J., Djureinovic, D., Takanen, J. O., Hober, S., Alm, T., Edqvist, P. H., Berling, H., Tegel, H., Mulder, J., Rockberg, J., Nilsson, P., Schwenk, J. M., Hamsten, M., von Feilitzen, K., Forsberg, M., Persson, L., Johansson, F., Zwahlen, M., von Heijne, G., Nielsen, J. & Ponten, F. (2015) Proteomics. Tissue-based map of the human proteome, *Science.* **347**, 1260419.

2. Consortium, G. T. (2013) The Genotype-Tissue Expression (GTEx) project, *Nat Genet.* **45**, 580-5.

3. Yan, L., Guo, H., Hu, B., Li, R., Yong, J., Zhao, Y., Zhi, X., Fan, X., Guo, F., Wang, X., Wang, W., Wei, Y., Wang, Y., Wen, L., Qiao, J. & Tang, F. (2016) Epigenomic Landscape of Human Fetal Brain, Heart, and Liver, *J Biol Chem.* **291**, 4386-98.

4. Bolger, A. M., Lohse, M. & Usadel, B. (2014) Trimmomatic: a flexible trimmer for Illumina sequence data, *Bioinformatics.* **30**, 2114-20.

5. Kim, D., Pertea, G., Trapnell, C., Pimentel, H., Kelley, R. & Salzberg, S. L. (2013) TopHat2: accurate alignment of transcriptomes in the presence of insertions, deletions and gene fusions, *Genome Biol.* **14**, R36.

6. Anders, S., Reyes, A. & Huber, W. (2012) Detecting differential usage of exons from RNA-seq data, *Genome research.* **22**, 2008-17.

7. Lei, L., Cao, W., Liu, L., Das, U., Wu, Y., Liu, G., Sohail, M., Chen, Y. & Xie, J. (2018) Multilevel Differential Control of Hormone Gene Expression Programs by hnRNP L and LL in Pituitary Cells, *Mol Cell Biol.* **38**.

8. Shen, S., Park, J. W., Lu, Z. X., Lin, L., Henry, M. D., Wu, Y. N., Zhou, Q. & Xing, Y. (2014) rMATS: robust and flexible detection of differential alternative splicing from replicate RNA-Seq data, *Proc Natl Acad Sci U S A.* **111**, E5593-601.

9. Xie, J. Y. & McCobb, D. P. (1998) Control of alternative splicing of potassium channels by stress hormones, *Science.* **280**, 443-446.

10. Xie, J. & Black, D. L. (2001) A CaMK IV responsive RNA element mediates depolarization-induced alternative splicing of ion channels, *Nature.* **410**, 936.

11. Trabzuni, D., Ramasamy, A., Imran, S., Walker, R., Smith, C., Weale, M. E., Hardy, J., Ryten, M. & North American Brain Expression, C. (2013) Widespread sex differences in gene expression and splicing in the adult human brain, *Nat Commun.* **4**, 2771.

12. Liao, Y., Smyth, G. K. & Shi, W. (2014) featureCounts: an efficient general purpose program for assigning sequence reads to genomic features, *Bioinformatics.* **30**, 923-30.

13. Robinson, M. D., McCarthy, D. J. & Smyth, G. K. (2010) edgeR: a Bioconductor package for differential expression analysis of digital gene expression data, *Bioinformatics.* **26**, 139-140.

14. Sohail, M., Zhang, M., Litchfield, D., Wang, L., Kung, S. & Xie, J. (2015) Differential expression, distinct localization and opposite effect on Golgi structure and cell differentiation by a novel splice variant of human PRMT5, *Biochim Biophys Acta.* **1853**, 2444-52.

15. Huang da, W., Sherman, B. T. & Lempicki, R. A. (2009) Systematic and integrative analysis of large gene lists using DAVID bioinformatics resources, *Nat Protoc.* **4**, 44-57.

16. Weinstein, J. N., Collisson, E. A., Mills, G. B., Shaw, K. R. M., Ozenberger, B. A., Ellrott, K., Shmulevich, I., Sander, C., Stuart, J. M. & Network, C. G. A. R. (2013) The Cancer Genome Atlas Pan-Cancer analysis project, *Nature Genetics.* **45**, 1113-1120.

17. Schlomm, T. (2020) Results of the CGC/TCGA Pan-Cancer Analysis of the Whole Genomes (PCAWG) Consortium, *Urologe.* **59**, 1552-1553.

18. Palmisano, A., Vural, S., Zhao, Y. & Sonkin, D. (2021) MutSpliceDB: A database of splice sites variants with RNA-seq based evidence on effects on splicing, *Hum Mutat.* **42**, 342-345.

19. Kolaskar, A. S. & Tongaonkar, P. C. (1990) A semi-empirical method for prediction of antigenic determinants on protein antigens, *FEBS Lett.* **276**, 172-4.

20. Larsen, M. V., Lundegaard, C., Lamberth, K., Buus, S., Lund, O. & Nielsen, M. (2007) Large-scale validation of methods for cytotoxic T-lymphocyte epitope prediction, *BMC Bioinformatics.* **8**, 424.

21. Abramson, J., Adler, J., Dunger, J., Evans, R., Green, T., Pritzel, A., Ronneberger, O., Willmore, L., Ballard, A. J., Bambrick, J., Bodenstein, S. W., Evans, D. A., Hung, C. C., O'Neill, M., Reiman, D., Tunyasuvunakool, K., Wu, Z., Zemgulyte, A., Arvaniti, E., Beattie, C., Bertolli, O., Bridgland, A., Cherepanov, A., Congreve, M., Cowen-Rivers, A. I., Cowie, A., Figurnov, M., Fuchs, F. B., Gladman, H., Jain, R., Khan, Y. A., Low, C. M. R., Perlin, K., Potapenko, A., Savy, P., Singh, S., Stecula, A., Thillaisundaram, A., Tong, C., Yakneen, S., Zhong, E. D., Zielinski, M., Zidek, A., Bapst, V., Kohli, P., Jaderberg, M., Hassabis, D. & Jumper, J. M. (2024) Accurate structure prediction of biomolecular interactions with AlphaFold 3, *Nature.* **630**, 493-500.
